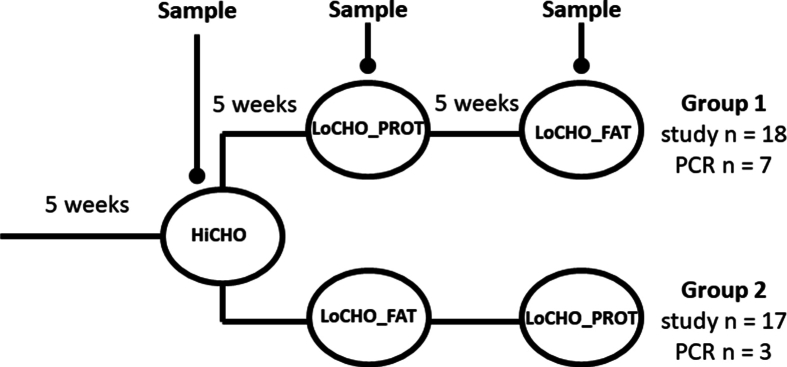# Immune-Modulating Effects of Low-Carbohydrate Ketogenic Foods in Healthy Canines

**DOI:** 10.1016/j.cdnut.2024.102128

**Published:** 2024-02-28

**Authors:** Selena K. Tavener, Matthew I. Jackson, Kiran S. Panickar

**Affiliations:** Science and Technology Center, Hill’s Pet Nutrition, Inc., Topeka, KS, United States

**Keywords:** immune, renal, inflammation, PCR, histopathology, canines

## Abstract

**Background:**

Ketogenic foods limit digestible carbohydrates but contain high fat, and have antioxidant and anti-inflammatory effects as well as improving mitochondrial function. β-Hydroxybutyrate (BHB), 1 of the ketone bodies, reduces the proinflammatory NLR family pyrin domain containing 3 inflammasomes, as well as chemokines in cultures.

**Objectives:**

We assessed the immune-modulating effects of 2 low-carbohydrate (LoCHO) foods varying in protein and fat and compared their effects with a food replete with high-carbohydrate (HiCHO) in healthy canines.

**Methods:**

Dogs were fed control food [HiCHO; ketogenic ratio (KR: 0.46) followed by LoCHO_PROT (KR: 0.97), then LoCHO_FAT (KR: 1.63) or LoCHO_FAT followed by LoCHO_PROT. Each food was fed for 5 wk, with collections in the 5th wk; 15 wk feeding total. Gene expression for circulating inflammatory cytokines from 10 dogs was assessed using the Canine RT^2^ Profiler polymerase chain reaction array, and fold changes were calculated using the ΔΔCt method.

**Results:**

LoCHO_FAT significantly increased circulating β-hydroxybutyrate compared with both HiCHO and LoCHO_PROT. When compared with HiCHO, there was a significant decrease in several proinflammatory cytokines/chemokines in LoCHO_PROT and LoCHO_FAT groups, including chemokine (C-C motif) ligand (CCL)1, CCL8, CCL13, CCL17, CCL24, chemokine (C-X3-C motif) ligand 1, chemokine (C-X-C motif) receptor 1, Interleukin-10 receptor alpha ((IL)-10RA), IL-1 receptor antagonist, IL-5, and secreted phosphoprotein 1 (all *P* < 0.05). Interestingly, a subset of inflammatory proteins that decreased in LoCHO_PROT but not in LoCHO_FAT included IL-33, IL-6 receptor, IL-7, IL-8, Nicotinamide phosphoribosyltransferase, and tumor necrosis factor (TNF) receptor superfamily member 11B. In contrast, the decrease in inflammatory markers in LoCHO_FAT, but not in LoCHO_PROT, included complement component 5, granulocyte colony-stimulating factor or G-CSF, interferon-γ, IL-3, IL-10RB, IL-17C, Tumor necrosis factor superfamily (TNFSF)13, TNFSF13B, and TNFSF14. Decreased concentrations of selected cytokines indicate that both low-carbohydrate foods exert an anti-inflammatory effect and provide a strong rationale for testing their efficacy in dogs with inflammatory conditions.

**Conclusions:**

Both LoCHO_PROT and LoCHO_FAT foods might be important as part of immune-modulating therapeutic nutritional strategies to reduce inflammation to maintain health in canines. Our study identifies several inflammatory genes that are reduced when fed ketogenic food that were not previously reported.

## Introduction

Ketogenic diets replace digestible carbohydrates with fat [1] to create foods that are low in carbohydrates (LoCHOs) and support a state of nutritional ketosis. Ketogenic diets were originally employed to reduce seizure incidence in childhood refractory epilepsy [2]. Food selection can influence metabolic predilection toward macronutrient metabolism [3]. Human subjects consuming LoCHO diets and ketogenic fats [e.g., those containing triglycerides of medium-chain (MCT) fatty acids] undergo a “metabolic switch,” resulting in an increased reliance on fat catabolism for energy metabolism [4]. The same phenomenon has recently been demonstrated in dogs [5]. In the context of acute viral infection, reduced capacity for metabolic switching to ketogenic metabolism results in impaired immune cell function, and the predominant ketone body β-hydroxybutyrate (BHB) restores cytokine production and endogenous antioxidant status of T-cells [6]. Although ketones were proposed to act by providing additional anabolic substrates, in that report, metabolic switching was independent of nutrient intake. The antioxidant effects of the ketogenic diet by reducing reactive oxygen species or limiting DNA damage have been reported in other contexts as well [7,8]. Switching to ketogenic metabolism has been explored for potential therapeutic value in managing inflammatory sequelae [9], and the impact of diet on immune function, including the role of LoCHO diets, has recently been reviewed [10].

The immunologic impact of ketogenic diets and ketones per se are intriguing and intertwined with aspects of the emerging space of immunometabolism, characterized by the cross-talk between metabolic regulation and immune function [11]. Ketogenic diets decrease anti-lymphangiogenic immune cells to decrease edema by improving the proliferation and metabolism of lymphatic endothelial cells [12] and also decrease concentrations of colonic innate immune cells [13] and T helper (Th) 17 cells [14] to improve colitis. Mechanistically, BHB is a histone-deacetylase inhibitor [15] that inhibits the NLR family pyrin domain containing 3 signaling to decrease inflammation [16]. It also decreases concentrations of IL-15 and the ratio of M1/M2 macrophage phenotype in adipose tissue [17]. In the BV-2 microglial cell line, BHB significantly attenuated the LPS-induced increase in proinflammatory IL-17 and also increased the concentrations of IL-10, an anti-inflammatory cytokine, inducing a polarization toward M2 anti-inflammatory phenotype [18]. The neuroprotective effect of BHB, mediated by its actions on hydroxy-carboxylic acid receptor 2, in a rodent model of stroke has been reported [19]. BHB has also been reported to upregulate Matrix metallopeptidase 2 generation to subsequently attenuate glomerulosclerosis in diabetic rats [20]. On the contrary, inflammatory status influences the metabolic phenotype. Postprandial circulating LPS endotoxin originating from gut commensal bacteria in concert with insulin stimulates adipose tissue macrophages to produce IL-10, which suppresses gluconeogenesis to improve glucose tolerance [21], whereas, at the same time, exposure to proinflammatory LPS endotoxin inhibits hepatic ketogenesis [22].

Domesticated dogs are susceptible to inflammatory conditions, such as chronic colitis, atopic dermatitis (AD) [23], and rheumatoid arthritis [24]. Although several of the studies on the beneficial effects of ketogenic foods have been reported in humans and rodent models, the immunologic response of dogs to ketogenic foods is not well characterized, particularly in the context of reducing dietary carbohydrates by replacing carbohydrate energy with protein (LoCHO_PROT) compared with fat (LoCHO_FAT). We recently reported that LoCHO_PROT and LoCHO_FAT are not equivalent in the degree to which they induce metabolic switching to ketogenesis in dogs; nutritional ketosis is induced to a greater extent from carbohydrate restriction with replacement by fat energy [5]. Ketogenesis is qualitatively similar between humans and canines, with a similar acetoacetate:BHB ratio and urinary ketone body excretion profile [25] as well as similar patterns of BHB use as fuel by the brain, heart, and kidney [[26], [27], [28]]. However, dogs are relatively resistant to fasting-induced ketosis Crandall [25]. Carbohydrate restriction-induced ketosis [29,30] mostly achieves concentrations of circulating BHB not considered ketotic in humans [31] and appears to have a periphery primarily reliant on nonesterified fatty acid catabolism for fuel [32]. Therefore, the degree to which carbohydrate restriction-induced nutritional ketosis in dogs may be accompanied by immunologic changes that occur in other species is not readily apparent.

Although there is evidence to support the anti-inflammatory effects of ketogenic foods, studies investigating the effects of ketogenic foods on the cytokine and chemokine profile in canines are scarce. To gain insight on this topic, a pilot study analyzing the immunomodulatory potential of LoCHO foods relative to a standard canine adult food replete with carbohydrates was assessed from a subset of samples in the aforementioned trial [5] in which each subject was fed each food in a crossover design. The results show that in addition to effects on circulating leukocytes, both LoCHO_PROT and LoCHO_FAT foods decrease several proinflammatory cytokines/chemokines. Taken alongside the metabolic effects previously reported from this trial [5], the results of the current report indicate that nutritional ketosis may link immunologic and metabolic phenomena in dogs to modulate immunometabolism.

## Methods

For PCR analyses reported here, 10 canine subjects were randomly selected from a larger population (*n* = 35) that enrolled in a previously reported trial [5]. The trial was performed under approval by the Institutional Animal Care and Use Committee (Protocol #: FP885.1.1.0-A-C-D-ADH-MULTI-112-MULTI), as well as Hill’s Pet Nutrition Animal Welfare Committee. The subset of 10 subjects selected was not significantly different from the larger set of subjects based on body weight and age (mean ± SE for all subjects from the trial compared with the subset selected: bodyweight 10.3 ± 0.3 compared with 10.1 ± 0.5 kg; age 7.1 ± 0.5 compared with 7.7 ± 0.8 y). This study was a prospective randomized crossover trial enrolling healthy adult beagle-breed dogs. This breed is not inordinately prone to seizures or inflammatory conditions relative to other breeds. Animal demographics of the entire study population and those selected for PCR analyses are reported in the supplementary information (Supplemental Table 1). Of the 10 subjects selected for PCR analyses, 7 dogs (2 neutered males and 5 spayed females) were in group 1 of the crossover design, and 3 subjects (2 spayed females and 1 neutered male) were in group 2. None of these 10 subjects were cohoused overnight; some were part of a larger housing unit and had free access to each other in the daytime during socialization and play.

All subjects consumed each of the 3 foods to allow for responses to foods to be compared within the subject. This repeated measures design permits dependent sample analyses and thus increases the power of the study to detect differences relative to a complete randomized design that generates independent samples. Subjects were randomly assigned to 2 groups based on demographics of age, weight, and gender to receive the high carbohydrate (HiCHO) food followed by the LoCHO foods in varying order. Study design and sampling points are depicted in Supplemental Figure 1. The wash-in food for both groups was HiCHO followed by either LoCHO_PROT before LoCHO_FAT or LoCHO_FAT before LoCHO_PROT (total *n* =35 divided into the 2 sequences of diet provision). Each food was fed for 5 wk, with collections in the 5th wk; 15 wk feeding total. There was no separate washout food between the sampling points in the crossover; however, there were 5 wk during each period for dogs to physiologically acclimate to each study food.

**FIGURE 1:**
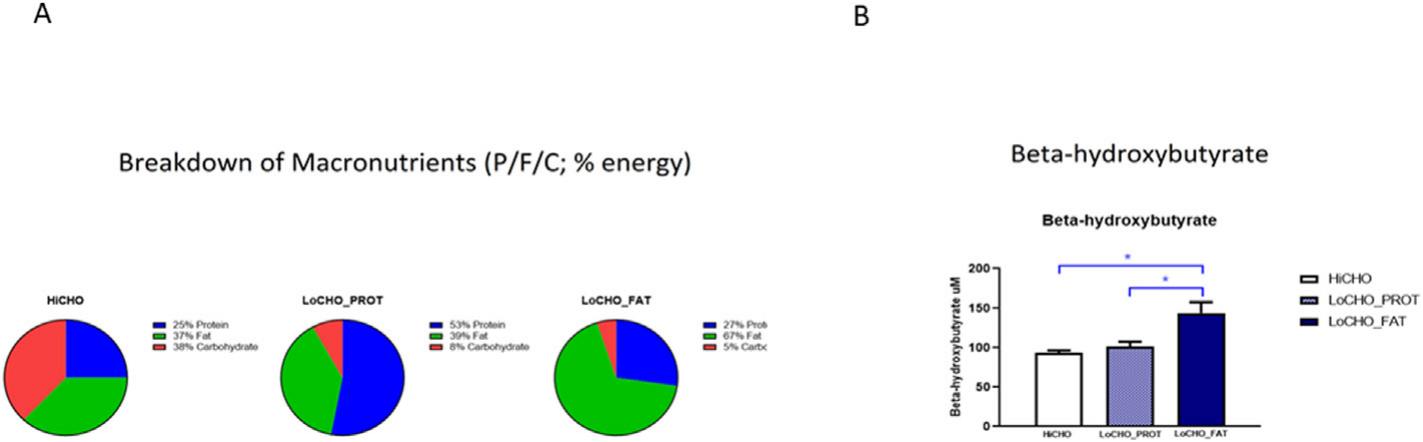
(A) A pie chart depicting the breakdown of macronutrients in the foods. Dogs (*n* = 35) were fed HiCHO food, which had a ketogenic ratio (KR) of 0.46; low carbohydrate with higher protein (LoCHO_PROT): KR = 0.97, and low carbohydrate with higher fat (LoCHO_FAT): KR = 1.63. (B) Circulating concentrations of β-hydroxybutyrate were increased significantly with LoCHO_FAT food but not with LoCHO_PROT when compared with HiCHO. Data are shown as mean ± SEM in both LoCHO diets and HiCHO. ∗*P* < 0.05. HiCHO, high carbohydrate; P/F/C, protein/fat/carbohydrate; SEM, standard error of the mean.

The food consisted of the following macronutrient compositions (protein/fat/carbohydrate; percentage energy, Figure 1A): HiCHO (25/37/38); LoCHO_PROT (53/39/8); and LoCHO_FAT (27/67/5). Further, the LoCHO_FAT food contained sources of MCT fat to bolster metabolic switching [4] and omega-3 PUFA, as these long-chain fats have been shown to be reduced by a ketogenic diet in an animal model [33]. Foods were analyzed for nutrients by the Association of Official Analytical Chemists methods (described in [5]).

All dogs were provided opportunities for daily exercise and interaction together in large groups (∼20 dogs) in outdoor grassy areas but were pair-housed overnight for sleeping arrangements. During the trial, dogs resided in their preferred housing arrangement, as previously determined by the colony veterinarian’s assessment of temperament and social interactions. Dogs were fed once daily at individual electronic feeders where each pet (through a radio frequency identification chip reader) was individually given access to a study food for 1 h in an amount calculated to maintain body weight. These electronic feeders recorded food intake (grams/day) for each dog. Dogs were fed to maintain body weight from the start of the study. One dog was removed from the study in the final portion of the crossover because of the development of chronic gastroenteritis unrelated to study foods [later diagnosed by the colony veterinarian as having inflammatory bowel disease (IBD)]; this dog was not included in the statistical analyses. All other dogs returned to the colony healthy after the study. Only noninvasive procedures were performed. Fasted blood collections were performed under approved protocols. Serum BHB as a marker of nutritional ketosis was assessed by enzymatic reaction. Clinical complete blood counts and biochemical analytes were analyzed by an in-house laboratory (Cobas 6000 series, c501 module; Roche Diagnostics). A subset of dogs was assessed for gene expression for inflammatory cytokines and receptors from whole blood using Canine RT^2^ Profiler PCR array (*n* = 10), and samples from this subset were analyzed from periods where these dogs were eating each food to allow for dependent (paired) sample analysis.

### Blood collection and RNA extraction

Gene expression was measured in blood collected in PAXgene RNA blood tubes (Qiagen). Total RNA was isolated using the PreAnalytix PAXgene blood miRNA kit (Qiagen). The Agilent RNA 6000 Nano kit was used to measure the integrity of total RNA using the Agilent Bioanalyzer 2100 (Agilent). The Qubit 3.0 Fluorometer was then used to measure the concentration of total RNA using the Qubit RNA BR Assay kit (Thermo Fisher). cDNA synthesis was then performed using the RT^2^ First Strand kit (Qiagen).

### Pathway-focused gene expression analysis

Changes in gene expression were analyzed using the Canine RT^2^ Profiler PCR Array Dog Inflammatory Cytokines and Receptors array platform (Qiagen) following the manufacturer’s protocol, as previously described [34]. Briefly, each 96-well array plate consisted of a focused panel of 84 genes. The last row of the array plate included the choice of 5 housekeeping genes for normalization, a specific well to detect genomic DNA contamination, reverse transcription control wells to monitor the efficiency of the reverse transcription reaction, and positive PCR control wells to test the efficiency of the PCR reaction. The total RNA input for each sample used for reverse transcription was 200 ng, and a components mix, including the cDNA, reverse transcribed from total RNA, was prepared and loaded onto the plate. The cycling program was set to 95°C for 10 min for the Hold Stage, followed by 40 cycles of 95°C for 15 s and 60°C for 60 s. A defaulted melting curve analysis was also performed to verify the specificity of the PCR analyzed using the ViiA7 Real-Time PCR instrument (Thermo Fisher). The Ct cutoff was set to 35 to be biologically meaningful. Data for the present study was normalized to β2-microglobulin (B2M) as the housekeeping gene as B2M showed the least amount of variance in cycle threshold (Ct) values between groups.

### Statistical analysis

For the PCR array analysis, the raw Ct values were uploaded to the data analysis web portal at https://geneglobe.qiagen.com/us/analyze. Samples were assigned to controls and test groups. Ct values were normalized to the housekeeping gene (B2M), and this was included in each plate. Fold changes for the PCR arrays were calculated relative to control using the ΔΔCt method. Negative values indicate a downregulation, where the fold-regulation is the negative inverse of the fold-change. The following statistical analyses were performed in JMP version 16.0. (SAS Institute Inc., 1989–2022): *1*) Linear mixed model with subject as a random factor to account for the repeated sampling from dogs consuming each diet. *2*) Wilcoxon nonparametric dependent samples t-test to assess whether individual transcripts or clinical metrics differed pairwise between diet treatments on a per-subject basis. Significance was set at α = 0.05. The primary comparison was between LoCHO_PROT and LoCHO_FAT, with further comparisons made with the HiCHO food.

## Results

### Biochemical assessment

All diets were well-tolerated with no adverse reactions. As reported in Jackson [5], dogs consumed more calories when being fed the LoCHO foods relative to the HiCHO food. Further, the mean percent intake of the entire meal offering was 93%–99%; there were no aberrant clinical analyte values resulting from these foods and no significant differences between diets for blinded observational subjective stool firmness scores (e.g., no loose stools). The ketogenic ratios (KRs) of the foods were HiCHO (KR 0.46), LoCHO_PROT (KR 0.97), and LoCHO_FAT (KR 1.63). Despite only 8% carbohydrate as energy, the LoCHO_PROT food was not ketogenic; LoCHO_PROT did not significantly elevate serum BHB (101.2 ± 0.09 μM) above that of the HiCHO food containing 38% starch as energy (93.6 ± 0.09 μM, Figure 1B). In contrast, the LoCHO_FAT food was ketogenic and significantly increased BHB (143.8 ± 0.09 μM) relative to both LoCHO_PROT and HiCHO (Figure 1B, both *P* < 0.05). Serum glucose concentrations were not significantly different between any of the groups [5].

### Clinical blood markers

**FIGURE 2:**
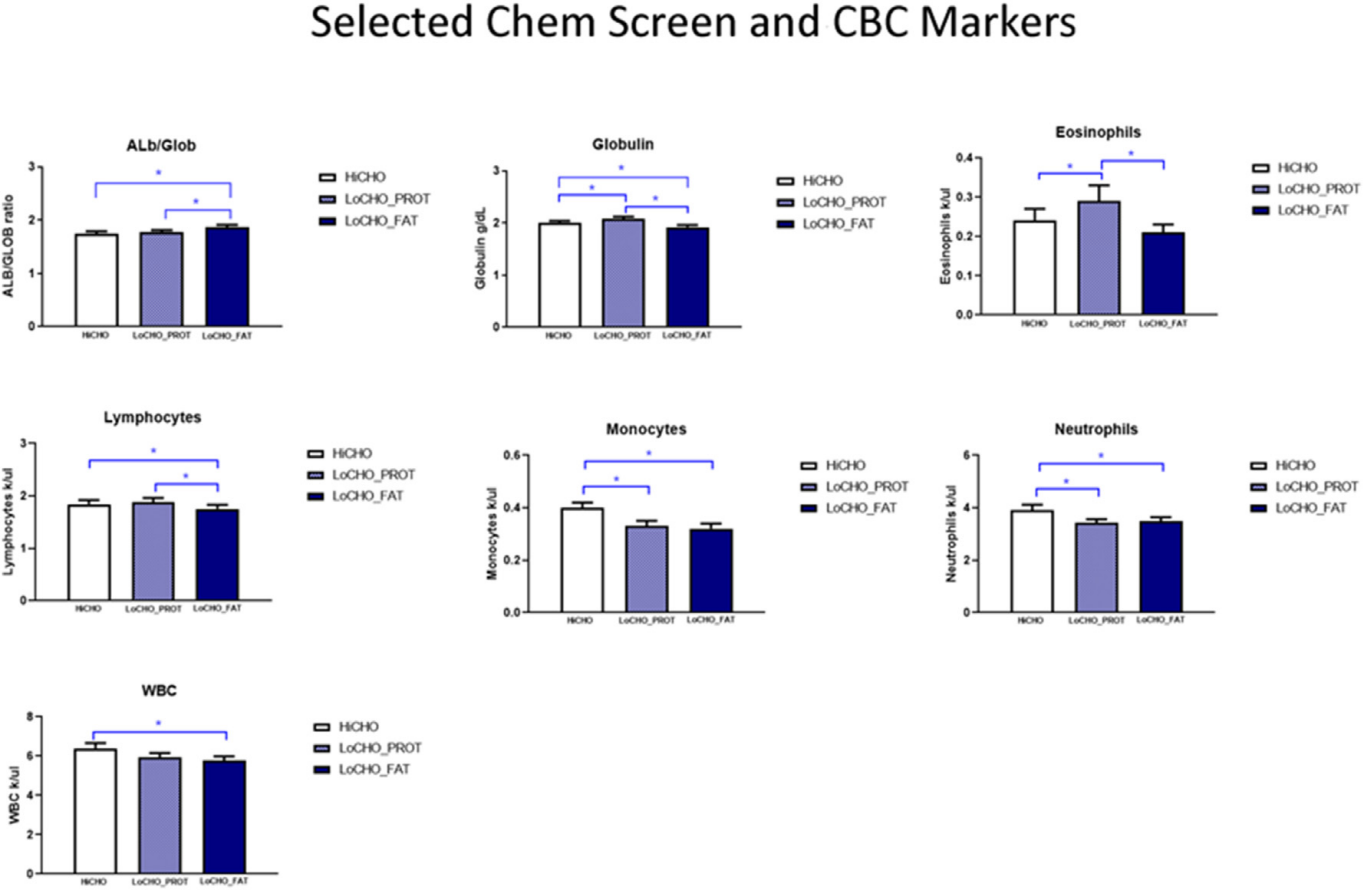
Effect of HiCHO, LoCHO_PROT, and LoCHO_FAT foods (*n* = 35) on laboratory measurements on complete blood chemistry values (CBC). Data are presented as mean + SEM (∗*P* < 0.05). ALB/GLOB, albumin/globulin; HiCHO, high carbohydrate; LoCHO_FAT, low carbohydrate_fat; LoCHO_PROT, low carbohydrate_protein; SEM, standard error of the mean; WBC, white blood cell.

The LoCHO_FAT food decreased absolute numbers of circulating lymphocytes and eosinophils as a percent of white blood cells (WBCs) while increasing neutrophils as a percent of WBC (Figure 2). The LoCHO_FAT food also decreased absolute numbers of circulating lymphocytes, monocytes, neutrophils, and total WBC compared with Control (CON). In contrast, LoCHO_PROT food increased eosinophils and lymphocytes but decreased monocytes and neutrophils as a percent of total WBC, as well as absolute numbers of monocytes compared with CON. LoCHO also decreased total circulating immunoglobin compared with LoCHO_PROT and CON, with the albumin/globulin ratio increased in LoCHO_FAT compared with LoCHO_PROT and CON. Indicating that the decrease in WBC was not accompanied by a general decrement in circulating blood cell production, both LoCHO_PROT, and LoCHO_FAT showed an apparent erythropoietic effect in that they increased red blood cells and hemoglobin; LoCHO_FAT also increased platelets relative to control. These markers were not different between the 2 LoCHO foods, except for platelets which were increased when dogs ate the LoCHO_FAT food compared with LoCHO_PROT.

### Pathway-focused gene expression

**FIGURE 3:**
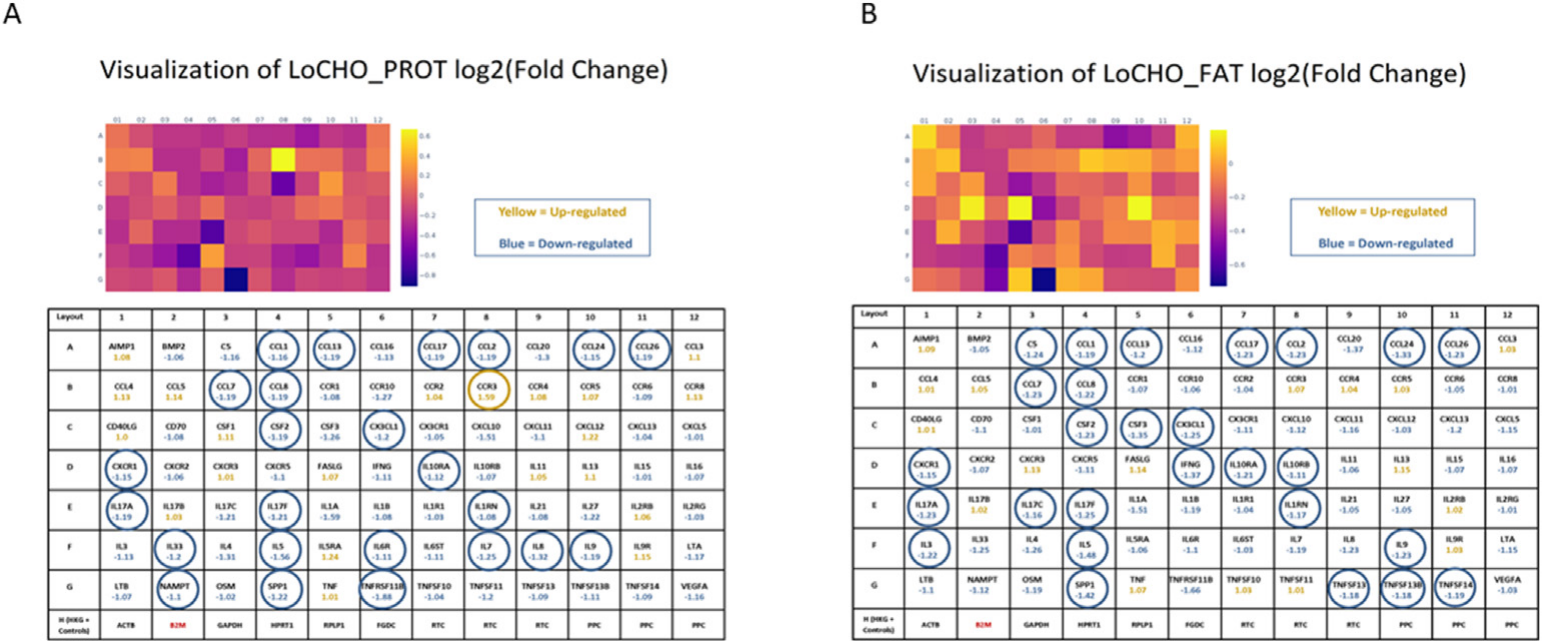
(A) Heat map showing the fold change in gene expression in LoCHO_PROT when compared with HiCHO. Gene expression studies were conducted in a subset of 10 dogs randomly selected from the 35 dogs (see text for details). Data are presented as mean fold change (ΔΔCt) normalized to β2-microglobulin (B2M), the housekeeping gene. Fold-change values >1 indicate an upregulation. Fold-change values <1 indicate a negative or downregulation, and the fold-regulation is the negative inverse of the fold-change (Qiagen). The individual squares in a heat map are scaled with a range of colors proportional to gene expression values. The figure below (Rows A–G) corresponds to the squares above, and each square indicates the mean values for that gene, with numerically positive values indicating up-regulation and negative values indicating down-regulation. Row H included the housekeeping genes ACTB, B2M, GAPDH, HPRT1, and RPLP1 in the assay. Other controls in row H included those for detection of genomic DNA contamination, reverse transcription control, and positive PCR control. Circled genes = *P* < 0.05. (B) It is the same as (A) but shows changes in LoCHO_FAT when compared with HiCHO. DNA, deoxyribonucleic acid; HiCHO, high carbohydrate; LoCHO_FAT, low carbohydrate_fat; LoCHO_PROT, low carbohydrate_protein; ACTB, actin beta; GAPDH, Glyceraldehyde 3-phosphate dehydrogenase; HPRT1, hypoxanthine phosphoribosyltransferase 1 and RPLP1, Ribosomal Protein Lateral Stalk Subunit P1.

We assessed the gene expression profile using the canine pathway-focused inflammatory PCR array panel and compared gene expression in LoCHO_PROT with HiCHO and also LoCHO_FAT with HiCHO. In general, there was a statistically significant decline in the expression of 24 circulating proinflammatory cytokines/chemokines in LoCHO_PROT when compared with HiCHO (Figure 3A). Of the 24 genes, there was a statistically significant decrease in 6 genes in dogs fed LoCHO_PROT, but not in LoCHO_FAT when compared with HiCHO, including IL-33, IL-6 receptor, IL-7, IL-8, Nicotinamide phosphoribosyltransferase, and TNF receptor superfamily member 11B (all *P* < 0.05; Figure 3A).

**FIGURE 4:**
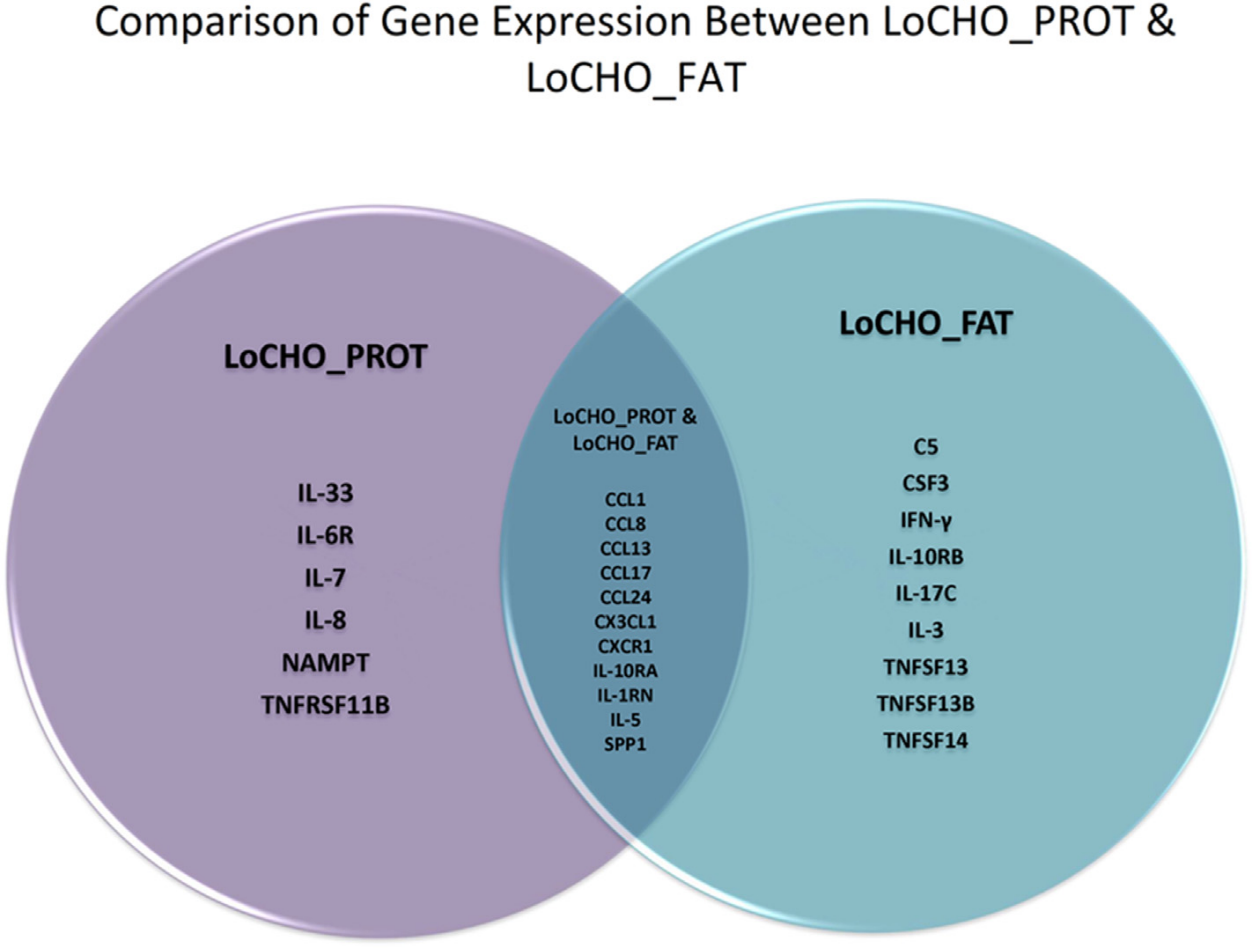
Venn diagram (*n* = 10) representing significant down-regulation of cytokines/chemokines observed in either LoCHO_PROT or LoCHO_FAT as well as significantly decreased expression in both diets when compared with the control food (HiCHO, *P* < 0.5). CCL, chemokine (C-C motif) ligand; CX3CL1, chemokine (C-X3-C motif) ligand 1; CXCR1, chemokine (C-X-C motif) receptor 1; HiCHO, high carbohydrate; IL-6R, interleukin 6 receptor; LoCHO_FAT, low carbohydrate_fat; LoCHO_PROT, low carbohydrate_protein; NAMPT, nicotinamide phosphoribosyltransferase; SPP1, secreted phosphoprotein 1; TNFRSF11B, tumor necrosis factor receptor superfamily member 11B; TNFSF13, tumor necrosis factor superfamily member 13; IL-10RA, Interleukin-10 receptor alpha; IL-10RB, Interleukin-10 receptor beta.

These genes, although also decreased with LoCHO_FAT when compared with HiCHO, were not statistically significant (Figure 3B). In addition, there was a decrease in 9 inflammatory markers in LoCHO_FAT, but not in LoCHO_PROT, including complement component 5 (C5), CSF3 (granulocyte colony-stimulating factor or G-CSF), interferon-gamma (IFN-γ), IL-3, IL-10RB, IL-17C, TNF superfamily (TNFSF) member 13 (TNFSF13), TNFSF13B, and TNFSF14 (all *P* < 0.05, Figure 3B). Interestingly, there was a statistically significant decrease in the same 13 proinflammatory cytokines/chemokines in both LoCHO_PROT and LoCHO_FAT when compared with HiCHO food. The genes that were statistically decreased in both included chemokine (C-C motif) ligand (CCL) (CCL1), CCL8, CCL13, CCL17, CCL24, chemokine (C-X3-C motif) ligand 1, Chemokine (C-X-C motif) receptor 1, IL-10RA, IL-1 receptor antagonist, IL-5, and secreted phosphoprotein 1 (all *P* < 0.05; Venn diagram in Figure 4). In contrast, there was a significant increase in chemokine (C-C motif) receptor 3 (CCR3), a receptor for several chemokines, in LoCHO_PROT but not in LoCHO_FAT when compared with control (*P* < 0.05).

## Discussion

Our study shows decreased circulating concentrations of selected cytokines when fed LoCHO_PROT or LoCHO_FAT foods when compared with HiCHO in healthy canines. This indicates the ability of both low-carbohydrate foods to exert an anti-inflammatory effect and provides a strong rationale for testing its efficacy in dogs with inflammatory conditions. Although the profile of inflammatory markers that were decreased with either LoCHO_PROT or LoCHO_FAT is similar for a selected set of cytokines, there is also a distinct difference in the effects of these 2 foods on gene expression. In contrast to the decline in other cytokines/chemokines, LoCHO_PROT, but not LoCHO_FAT, significantly increased CCR3 concentrations compared with HiCHO. CCR3 is a G-protein coupled receptor for many eotaxins, including CCL11 and CCL26, and also a receptor for CCL13. CCR3/eotaxin complex can promote B-cell apoptosis and decrease IgE production [35] and thus may be involved in reducing an allergic response. Given the effects of LoCHO foods in reducing concentrations of selected inflammatory markers, it is conceivable that these foods will be beneficial in canine disorders where inflammation plays a critical role. Our results, in general, are consistent with the anti-inflammatory effects of ketogenic foods in humans, as discussed below, but studies on their effects on canine inflammation are scarce.

### Obesity

Our results are consistent with the anti-inflammatory effects of ketogenic diets that have been reported, although the panel of inflammatory markers assessed and reported in human studies is small. Alkhorayef et al. [36] reported that BHB supplementation of BHB to Saudi females with obesity for 8 wk resulted in a decrease in the circulating concentration of IL-1β, a cytokine. Lorenzo et al. [37] reported patients who were overweight or obese who went through a weight reduction program either through a very low-calorie ketogenic diet or a standard, balanced hypocaloric diet or bariatric surgery. They showed that a very low-calorie ketogenic diet showed the most pronounced effect in reducing circulating cytokine concentrations, including IL-11, IL-12, IL-2, IFN-γ, IFN-β, and Pentraxin-3. Paoli et al. [38] investigated the effects of a ketogenic diet on competitive natural bodybuilders. When compared with a Western diet fed for 2 mo, the ketogenic diet showed a reduction in circulating concentrations of cytokines, including IL-1β, IL-6, and TNF-α. Bosco et al. [39] investigated the effect of the short-term ketogenic diet (7 d) on reducing inflammation in overweight divers (*n* = 6) during a hyperoxic dive. When compared with baseline, circulating markers of inflammation, including IL-1β, IL-6, and TNF-α, were significantly reduced with the ketogenic diet as assessed after the dive performed at 7 d after the ketogenic diet. In contrast, Myette-Côté et al. [40] did not observe a decrease in plasma inflammatory markers in patients with mild cognitive impairment who were fed keto-MCT (30 g/d) for 6 mo when compared with a placebo drink. They observed an increase in circulating IL-8, and the reason for this is unknown. Similarly, Cipryan et al. [41] also did not see considerable changes in markers of inflammation, including adiponectin and IL-6, in healthy subjects who were fed a very low-carbohydrate high-fat diet for 4 wk. Whether the discrepancy in the result is because of the different KRs of different ketogenic diets is not clear, but it is a possibility.

### Dermatitis

Canine AD (CAD) is an inflammatory chronic skin condition characterized predominantly by pruritus and erythema. Given the multi-factorial nature of the pathogenesis of AD, it is not surprising that Th1, Th2, Th17, and Th22 lymphocytes and the cytokines they release, as well as increased IgE production, have all been implicated in dermatitis in humans [42], rodents [43,44], and canines [[45], [46], [47]]. In our study, LoCHO_FAT food significantly decreased CSF3, a cytokine produced by macrophages and epithelial cells (keratinocytes), which promotes the activation of dendritic cells and neutrophils. Pastore et al. [48] reported an enhanced production of CSF2 by keratinocytes in a small study in humans with chronic AD lesions. Jassies-van der Lee et al. [49] reported clusters of differentiation 4 and clusters of differentiation 8 T-cell subsets of lesional and nonlesional CAD skin, using explant cultures, produced IFN-γ, IL-13, and IL-22. LoCHO_FAT, but not LoCHO_PROT, significantly decreased IFN-γ concentrations. The role of complement activation and its effects on dermatitis is not clear. Complement C5a can degranulate mast cells and basophils, which can induce an allergic response. In a very early study, when assessed for complement and IgG deposits in the skin of humans with AD (*n* = 5), there were complement C5 deposits in 2 of the patients [50]. However, Kapp and Schöpf [51] did not detect any measurable amount of C5a in the blood of patients with mild to moderate AD, although complement component 3 (C3) was higher in patients with AD (complement C3b is a convertase that can induce cleavage of C5). Whether C5 plays a role in CAD is not known, but the innate immune system plays an important role in the effector phases of CAD [52]. Importantly, the ability of LoCHO_FAT to decrease C5 in canines may be beneficial. LoCHO_PROT significantly decreased IL-33 concentrations. Increased IL-33 is associated with chronic lesional skin of CAD [53]. Interestingly, both LoCHO_PROT and LoCHO_FAT significantly decreased IL-5 concentrations. IL-5 is a cytokine that is produced by mast cells and Th2 cells and is involved in an allergic response that is mediated by B-cells and eosinophils (see [54] for review) IL-5 is also a growth factor for B-cells. We have previously reported an increase in circulating IL-5 and IL-5RA mRNA in canines with dermatitis when compared with controls [55]. Given the importance of the IL-5/IL-5RA axis in canine dermatologic problems, it is conceivable that these conditions might benefit from LoCHO_FAT and/or LoCHO_PROT foods.

### Renal

Inflammation is one of the key contributors in the initiation and progression of acute as well as chronic kidney disease (CKD) in humans [56,57], and different cytokines may characterize acute or chronic kidney dysfunction [58]. Similarly, in canines, cytokines including IL-1 alpha, IL-1β, and transforming growth factor-β and the enzyme 5-lipoxygenase are increased in acute kidney injury (AKI) and CKD [59]. Further, canine end-stage renal disease, characterized by advanced glomerular sclerosis, interstitial inflammation, and fibrosis, is associated with the expression of increased inflammatory markers. Yhee et al. [60] reported significant fibrosis as well as increased expression of cytokines, including IL-1 and IL-6 concentrations, in renal tissue taken from dogs with end-stage renal disease. These studies implicate proinflammatory factors in renal dysfunction, and given the complexity of kidney dysfunction, it is conceivable that the underlying factors are multifactorial. Recognition of newer inflammatory signals, as well as newer therapeutic approaches, is imperative. In the current study, LoCHO_FAT food decreased complement C5, and blockade of C5 has been reported to attenuate renal failure, including C3 glomerulopathy, an abnormal complement activation, in mice [61]. Pretreatment with a C5-inhibiting monoclonal antibody in a mouse model of anti-myeloperoxidase IgG-induced glomerulonephritis significantly reduced glomerular crescent formation [62]. In humans, Ravulizumab, a monoclonal antibody to complement C5, has been approved for atypical hemolytic uremic syndrome and is being evaluated in early-phase and preclinical studies for reducing renal disorders [63]. Given the importance of complement-activated renal dysfunction, the ability of the LoCHO_FAT diet to decrease circulating C5 in canines may potentially offer newer nutritional therapeutic approaches to attenuate renal dysfunction.

Other cytokines that are also implicated in renal dysfunction and which decreased with LoCHO_FAT include IFN-γ, IL-3, IL-17C, and CSF-3. IFN-γ, a proinflammatory cytokine, contributes to the renal fibrotic process and progression to CKD in mice [64]. The mechanism by which LoCHO_FAT food reduced IFN-γ is not clear, although Lu et al. [65] reported a reduction in the nuclear factor of the kappa beta signaling pathway and IFN-γ with the ketogenic diet in a rat model of spinal cord injury. He et al. [66] reported an increase in IL-3 in renal tissue in a mouse model of AKI. Although investigating the role of septic shock after AKI in humans, IL-17 was the only cytokine significantly increased in peripheral blood mononuclear cells and clusters of differentiation 4-lymphocytes in patients with septic shock and AKI than controls [67], indicating the role of Th17 cells in kidney dysfunction. There is evidence to indicate that IL-33, a proinflammatory cytokine that mediates tissue inflammation, contributes to CKD and is also implicated in AKI, as well as in the progression of renal fibrosis [68,69]. Both LoCHO_PROT and LoCHO_FAT decreased IL-33, and their role in managing or attenuating kidney failure is warranted.

### Cancer

There is increasing evidence to indicate a link between inflammation and cancer. Inflammatory cells, including macrophages and neutrophils, are important in the initiation of cancer [70,71], possibly through their production of reactive oxygen species. It should, however, be noted that an elevated response of certain cytokines or chemokines plays a role in suppression or as a part of the therapeutic strategy, including IFN-α in humans for leukemia and IL-2 for metastatic renal cell carcinoma (see Conlon et al. [72]). Nevertheless, a reduction of critical or novel inflammatory mediators may contribute to the induction and progression of tumors [73]. Given that nutrition is an important component to decreasing a predisposition to cancer or is recommended as an adjuvant during cancer therapy, foods that can reduce proinflammatory cytokines or chemokines may improve prognosis.

LoCHO_FAT, but not LoCHO_PROT, significantly decreased complement C5. Increased C5 may play a role in the initiation of selected tumors. Using human colonic epithelial cells, Ding et al. [74] reported that C5a/C5a receptor 1, through activation of β-catenin, promoted colorectal tumorigenesis. Ortiz-Espinosa et al. [75] also reported a role for C5a in tumorigenesis. They reported that inhibition of C5a or C5a receptor 1 may lower the number of circulating tumor cells and the metastatic burden in a mouse lung metastasis model. These studies indicate an important role of C5 as a biomarker in tumor progression, and it is conceivable that a reduction of C5 by LoCHO_FAT may be beneficial to overall health during cancer therapy.

LoCHO_FAT also decreased TNFSF13, a proliferation-inducing member of the TNFSF, which is associated with poor prognosis, indicating its potential as a nutritional intervention strategy for cancer. TNFSF13 promoted multiple myeloma cell survival through its actions on Breg and Treg cells [76]. Garcia-Castro et al. [77] reported increased concentrations of TNFSF13 in more aggressive basal tumors in human breast carcinomas. LOCHO_FAT, but not LOCHO_PROT, decreased IL-17C concentrations. IL-17C is a proinflammatory cytokine and a member of the IL-17 family of cytokines. IL-17C is hypothesized to contribute to the pathogenesis of several infectious diseases as well as cancer [78]. IL-17C promotes lung inflammation and is present in human lung tumors, and Ritzmann et al. [79] showed that tumor growth was decreased in IL-17C deficient mice but not in wild-type mice after antiprogrammed cell death treatment. IL-17 is upregulated in the tissue as well as in the serum of glioma patients and is also hypothesized to increase the proliferation and migration of tumors in glioma [[80], [81], [82]]. In addition, Wainwright et al. [80] also reported the presence of IL-17 in a mouse model of malignant glioma. Whether IL-17 members play a role in canine cancer is not known, but a food that has the potential to reduce IL-17C may be important as more information is available on the role of cytokines in canine cancer development. Our study demonstrates that LoCHO_FAT and LoCHO_PROT have the potential to reduce several proinflammatory markers that are known to play a role in cancer initiation and progression.

### IBD

IBD is generally characterized by chronic inflammation of the gastrointestinal tract and may include pain, swelling, and diarrhea. Proinflammatory cytokines, including TNF-α, IL-6, and IL-23, are all known to play an important role in IBD in humans [83]. Several proinflammatory markers, including IL-3, IL-33, and IL-8, that were reduced by LoCHO_FAT or LoCHO_PROT have been reported to be associated with IBD. In our study, LoCHO_FAT significantly reduced circulating IL-3 concentrations. Although the role of IL-3 in IBD in canines is not known, it is predominantly produced by activated T-cells as it has growth factor-like effects on monocytes/macrophages, mast cells, and basophils [84]. Basophils and monocytes have been implicated in IBD in human samples, as assessed by the presence of monocyte chemotactic protein-3 expression, an activator of basophils and monocytes, in sites of active mucosal inflammation [85]. Interestingly, an increased concentration of monocytes is associated with a high degree of probability of relapse of IBD in patients during remission [86]. LoCHO_PROT, but not LoCHO_FAT, significantly reduced concentrations of IL-8 and IL-33, and both inflammatory markers are associated with IBD. Tamura et al. [87] report a modest increase in IL-8 in the colonic mucosa in dogs with lymphocytic-plasmocytic colitis when compared with healthy dogs, but this was not significant. Maeda et al. [88] reported a significantly higher concentration of C-X-C motif chemokine ligand 8 (IL-8) in the duodenal mucosa of dogs with IBD when compared with controls, and this increase was significantly correlated with a clinical severity score. Subsequently, Maeda et al. [89], using duodenal mucosal cultures from healthy and IBD dogs, showed an increased concentration of IL-8 in IBD when compared with controls when stimulated with a protease-activated-2 receptor agonist. Proteases are hypothesized to play a role in inducing an inflammatory response in the gastrointestinal tract. These studies indicate that IL-8 is an important contributor to IBD, and strategies to reduce their expression may be beneficial. IL-33 is another cytokine, and in some cases acts as a transcription factor, that is implicated in IBD in humans [90]. However, the role of IL-33 in the pathology of IBD is not clear as it can exert a proinflammatory effect and, in some instances, an anti-inflammatory effect as well [91,92]. LoCHO_PROT significantly decreased IL-33 concentrations. There is evidence to indicate that IL-33 can induce a pathogenic response in IBD in humans. IL-33 concentrations are increased in inflamed colonic mucosa and serum and correlated with disease activity in Crohn’s disease in humans [93]. In rodents, IL-33 treatment impaired epithelial barrier permeability in vitro and in vivo in an experimental model of colitis in mice [94]. The role of IL-33 in promoting intestinal fibrosis in a mouse model of Crohn’s disease-like ileitis has also been reported [95,96]. The pathogenesis of IBD is complex, and it is likely that multiple inflammatory signaling pathways are activated. Identification of newer candidates that may play an important role in IBD can lead to newer therapeutic strategies, including the recommendation of LoCHO foods for the management of the condition.

In summary, the results from our study demonstrate a potential anti-inflammatory effect of LoCHO foods in healthy canines. We identified several proinflammatory cytokines/chemokines and their receptors that are down-regulated, very likely because of decreasing digestible carbohydrates in canine foods as well as because of the combination of carbohydrates with varying concentrations of protein and fat. Increased concentrations of the proinflammatory chemokines and their receptors play an important role in the recruitment of T-cells, macrophages, and dendritic cells and subsequent inflammation. Based on the findings from our study it appears that ketogenic foods can exert an anti-inflammatory effect and thus provide a strong rationale for testing its efficacy in dogs with inflammatory conditions. The strength of our study is that it identifies several cytokines and chemokines affected by ketogenic food that were not previously reported in healthy dogs. It also provides a strong rationale for further elucidating the role of individual cytokines/chemokines in health and disease in canines. The limitations of our study would include a relatively smaller sample size as well as not further investigating if circulating protein concentrations correlate with the gene expression data. Taken together with previous findings showing that these same LoCHO foods induced metabolic changes, the current report bolsters the case that immunometabolism may be a viable therapeutic target in canines. Commercial LoCHO formulations are currently available for dog owners. Possible practical applications of this research include new nutritional recommendations for commercial LoCHO foods with future implications for the treatment of a broad spectrum of chronic health conditions.

## Author contributions

The authors’ responsibilities were as follows – SKT: conducted the research, analyzed the data, and wrote the paper; MIJ: designed the research, analyzed the data, and wrote the paper; KSP: conducted the research, provided essential reagents, analyzed the data, wrote the paper, and had primary responsibility for final content; and all authors: read and approved the final manuscript.

## Conflict of interest

SKT, MIJ, and KSP are employees of Hills Pet Nutrition Center, a Colgate-Palmolive Company.

## Funding

This work was funded by Hill’s Pet Nutrition Center, a Colgate-Palmolive Company.

## Data availability

Data will be made available upon request.

## Figures and Tables

**Figs1:**