# Supplementary material for: Immune-Modulating Effects of Low-Carbohydrate Ketogenic Foods in Healthy Canines
Source: Curr Dev Nutr. 2024 Feb 28;8(4):102128. doi: 10.1016/j.cdnut.2024.102128 (PMC10999821; doi:10.1016/j.cdnut.2024.102128)
Supplement: Multimedia component 1 [file mmc1.pdf]

Immune-modulating effects of low carbohydrate ketogenic foods in healthy canines
Selena K. Tavener

SUPPLEMENTAL TABLE 1

Subject ID	PCR Analysis Paper?	Diet Order Group	Gender	age (yr)	body weight (kg)	Housing Location	Breed
29	YES	1	Neutered Male	3.1	10.6	B5-11-12	Beagle
3	YES	1	Neutered Male	8.1	12	B7-53-18	Beagle
36	YES	1	Spayed Female	9.6	10.5	B5-21-15	Beagle
31	YES	1	Spayed Female	9.5	7.4	B6-42-08	Beagle
11	YES	1	Spayed Female	4.2	10.5	B7-52-13	Beagle
34	YES	1	Spayed Female	8.1	8.8	B7-53-19	Beagle
13	YES	1	Spayed Female	8.2	11.7	B7-62-14	Beagle
24	YES	2	Neutered Male	11.2	12.3	B5-23-05	Beagle
20	YES	2	Spayed Female	9.5	8.6	B5-13-06	Beagle
16	YES	2	Spayed Female	5.3	8.2	B5-23-04	Beagle
7	NO	1	Neutered Male	7	10.6	B5-12-15	Beagle
8	NO	1	Neutered Male	7.7	11.8	B6-31-12	Beagle
5	NO	1	Neutered Male	9	11.1	B6-31-19	Beagle
4	NO	1	Neutered Male	7.3	11.4	B6-32-02	Beagle
2	NO	1	Neutered Male	1.5	12.6	B6-41-10	Beagle
30	NO	1	Neutered Male	2	9.5	B6-44-15	Beagle
9	NO	1	Neutered Male	7	9.5	B7-52-10	Beagle
6	NO	1	Spayed Female	7.7	8.1	B5-23-03	Beagle
32	NO	1	Spayed Female	5.3	9	B6-44-04	Beagle
33	NO	1	Spayed Female	6.3	8.2	B7-54-15	Beagle
22	NO	1	Spayed Female	8.1	11.2	B7-63-10	Beagle
14	NO	2	Neutered Male	11.2	11.3	B5-21-13	Beagle
27	NO	2	Neutered Male	9.5	11.8	B5-21-20	Beagle
15	NO	2	Neutered Male	11.2	10.6	B5-23-17	Beagle
12	NO	2	Neutered Male	4.2	11.7	B5-23-18	Beagle
10	NO	2	Neutered Male	7.7	10.6	B5-24-09	Beagle
17	NO	2	Neutered Male	9.3	11.3	B6-31-14	Beagle
25	NO	2	Neutered Male	2.1	9	B6-43-17	Beagle
28	NO	2	Spayed Female	9.3	9.3	B5-14-02	Beagle
1	NO	2	Spayed Female	8.1	10.7	B5-21-09	Beagle
35	NO	2	Spayed Female	5.3	10.8	B5-23-20	Beagle
21	NO	2	Spayed Female	8.3	10.3	B6-32-12	Beagle
26	NO	2	Spayed Female	4.6	12.5	B7-52-09	Beagle
23	NO	2	Spayed Female	3.1	7.5	B7-53-07	Beagle
18	NO	2	Spayed Female	8.1	7.9	B7-54-14	Beagle
